# Autophagy Blockage Reduces the Incidence of Pancreatic Ductal Adenocarcinoma in the Context of Mutant *Trp53*

**DOI:** 10.3389/fcell.2022.785252

**Published:** 2022-03-16

**Authors:** Laura Mainz, Mohamed A. F. E. Sarhan, Sabine Roth, Ursula Sauer, Katja Maurus, Elena M. Hartmann, Helen-Desiree Seibert, Andreas Rosenwald, Markus E. Diefenbacher, Mathias T. Rosenfeldt

**Affiliations:** ^1^ Institute of Pathology, Julius-Maximilians-University of Würzburg, Würzburg, Germany; ^2^ Comprehensive Cancer Center Mainfranken, Julius-Maximilians-University of Würzburg, Würzburg, Germany; ^3^ Salk Institute for Biological Studies, San Diego, CA, United States; ^4^ Biocenter, Department of Biochemistry and Molecular Biology, Julius-Maximilians-University of Würzburg, Würzburg, Germany

**Keywords:** pancreatic cancer, autophagy, p53, metastasis, ATG7

## Abstract

Macroautophagy (hereafter referred to as autophagy) is a homeostatic process that preserves cellular integrity. In mice, autophagy regulates pancreatic ductal adenocarcinoma (PDAC) development in a manner dependent on the status of the tumor suppressor gene *Trp53*. Studies published so far have investigated the impact of autophagy blockage in tumors arising from *Trp53*-hemizygous or -homozygous tissue. In contrast, in human PDACs the tumor suppressor gene *TP53* is mutated rather than allelically lost, and TP53 mutants retain pathobiological functions that differ from complete allelic loss. In order to better represent the patient situation, we have investigated PDAC development in a well-characterized genetically engineered mouse model (GEMM) of PDAC with mutant *Trp53* (*Trp53* ^*R172H*^) and deletion of the essential autophagy gene *Atg7*. Autophagy blockage reduced PDAC incidence but had no impact on survival time in the subset of animals that formed a tumor. In the absence of *Atg7*, non-tumor-bearing mice reached a similar age as animals with malignant disease. However, the architecture of autophagy-deficient, tumor-free pancreata was effaced, normal acinar tissue was largely replaced with low-grade pancreatic intraepithelial neoplasias (PanINs) and insulin expressing islet β-cells were reduced. Our data add further complexity to the interplay between *Atg7* inhibition and *Trp53* status in tumorigenesis.

## Introduction

PDAC is the most frequent type of pancreatic cancer and has a 5-year survival rate between 5–10%. Autophagy promotes cellular homeostasis and is a process that involves cargo transport within specialized vesicles, so-called autophagosomes, to lysosomes for degradation. There is widespread belief that autophagy blockage is potentially a promising treatment option for PDAC, but its applicability is currently limited at best, due to its still incompletely understood effects on PDAC biology (Piffoux et al., 2021). Two critical determinants for the net outcome of autophagy impairment are the time point during tumorigenesis at which autophagy is modulated and the genetic background. There is emerging consensus that autophagy is dispensable at the early stages of tumor development but required for tumor maintenance. GEMMs that allow deletion of the essential autophagy regulating genes *Atg5* or *Atg7* have been instrumental in deciphering the complex relation of wild-type *Trp53* and autophagy in *de novo* tumor development from healthy embryonic tissue. Depending on the status of *Trp53*, autophagy either promotes or suppresses PDAC development (Rosenfeldt et al., 2013; Yang et al., 2014). However, in human PDAC, *TP53* is frequently mutated rather than allelically lost and TP53 mutants retain a certain degree of functionality with different pathobiological consequences (Saiki and Horii, 2014; Sabapathy and Lane, 2018). For this reason, we investigated the impact of autophagy blockage on *de novo* PDAC formation in the context of a mutant *Trp53* allele (*Trp53* ^*R172H*^) in mice.

## Methods

### Animal Experiments

All experiments were approved by the Government of Lower Franconia (Regierung Unterfranken) and carried out under the license number 2532-2-313. Mouse strains were described previously and in our experiments were of C57BL/6 background (Komatsu et al., 2005; Morton et al., 2010). FELASA guidelines were followed for animal maintenance and mice were housed in standard cages in pathogen-free facilities on a 12 h light/dark cycle with ad libitum access to food and water. Animals were monitored twice weekly for tumor formation and sacrificed once endpoint criteria were met (weight loss, weakness and inactivity).

### Tissue Preparation

After euthanasia, tissues were excised and fixed in 10% neutral buffered formalin for 24 h at room temperature. Fixed tissues were paraffin embedded and 4 µm sections were prepared for hematoxylin and eosin (HE) staining and immunohistochemistry.

### Immunohistochemistry

Immunohistochemical stains were performed as previously described (Rosenfeldt et al., 2012; Rosenfeldt et al., 2013). The following antibodies were used: ATG7 (D12B11) rabbit monoclonal (Cell Signaling Technologies, #8558, dilution 1:100, pretreatment TR pH 9.0), Insulin (Dako, #5064, dilution 1:100, pretreatment CS pH 6.0), LC3 mouse monoclonal 5F10 (Nanotools, #0231-100/LC3-5F10, dilution 1:100, pretreatment TR pH 9.0), SQSTM1/P62 rabbit polyclonal (Enzo, #BML-PW9860, dilution 1:500, pretreatment TR pH 6.1). ATG7 and LC3 were visualized with SuperVision 2 HRP (mouse/rabbit) (DCS Innovative Diagnostik-System, PD000POL), SQSTM1/P62 with HiDef Detection™ HRP Polymer System (Cell Marque, 954D-40) and Insulin with Histostain-Plus broad spectrum (Invitrogen, 859043). (TR = antigen retrieval with target retrieval antigen solution from Agilent DAKO, S2367; CS = target retrieval with citrate buffer).

To quantify the percentage of insulin positive islet cells, islet cells that showed an appreciable expression of insulin were counted manually in at least five randomly chosen islets of Langerhans per mouse and divided by the total number of islet cells (on average 345 islet cells/mouse).

### Genotyping

Genotyping was done as described by Morton et al. for *Pdx1-Cre*; *KRas* ^*G12D*^; *Trp53* ^*R172H*^ and by Komatsu et al. for Atg7^fl^ (Komatsu et al., 2005; Morton et al., 2010).

### Targeted Sequencing

Formalin-fixed paraffin-embedded (FFPE) tissue specimens were microdissected to isolate the tissue of interest, i. e. PDAC in tumor-bearing mice and pancreatic tissue with abundant PanINs in non-tumor-bearing animals. Subsequently, genomic DNA was extracted with the Maxwell RSC Blood DNA Kit after a pre-treatment with a THG1-Thioglycerol/incubation buffer mix for 10 min at 80°C and subsequent incubation with proteinase K at 65°C overnight (Promega, #AS1400). Targeted deep sequencing using a customer designed Ion Ampliseq panel of Thermo Fisher including exonic regions of *Trp53* was performed to assess copy number loss of the wildtype *Trp53* allele. Cellularity of neoplastic cells (PanINs in non-tumor-bearing mice or tumor cells in mice with PDAC) was estimated by three experienced pathologists (AR, EMH, MTR).

### Statistical Analysis

Statistical analyses were carried out using IBM SPSS Statistics version 21 for Windows. The relevant statistical tests are mentioned in the figure legend.

## Results

**FIGURE 1:**
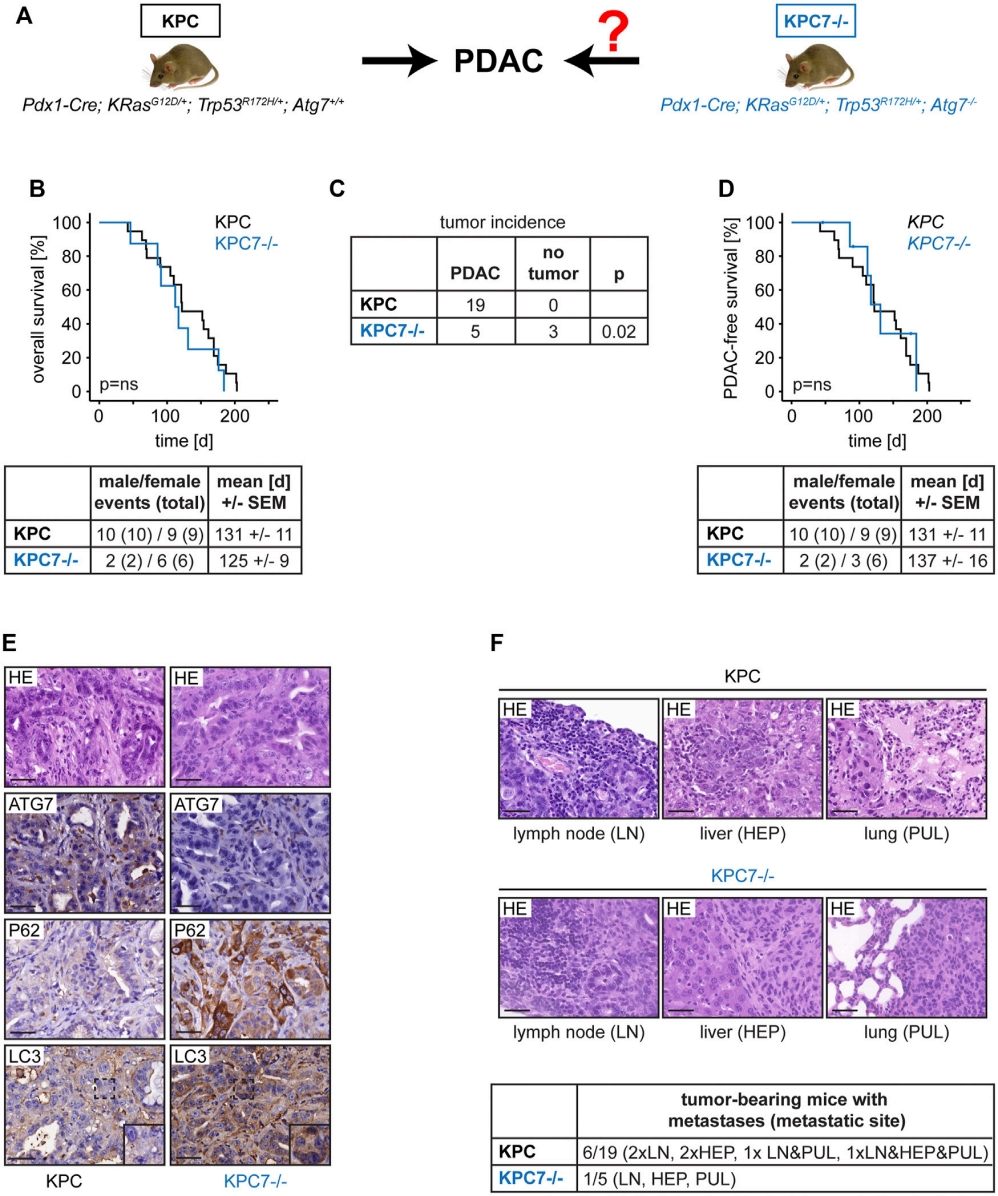
Effects of autophagy blockage on PDAC development in the context of mutant Trp53. **(A)** Mouse cohorts and scientific question. **(B)** Overall survival and cohort composition. **(C**) Tumor incidence of the indicated cohorts. **(D)** PDAC-free survival and cohort composition. **(E)** Representative PDAC histology and immunohistochemistry as indicated. Scale bars represent 40 µm. **(F)** Metastases frequency and exemplary histology. Scale bars represent 40 µm. Statistics: Log-Rank Test **(B and D)**, Fisher’s Exact Test **(C)**, ns = not significant.

*Pdx1-Cre; KRas* ^*G12D/+*^ *; Trp53* ^*R172/+*^ (=KPC) mice are a well-characterized model of PDAC development with genetic recombination occurring in healthy pancreatic tissue during embryogenesis (Morton et al., 2010). These mice were crossed to animals that contain floxed alleles of *Atg7* (*Atg7* ^*fl/fl*^) to create a cohort (*Pdx1-Cre; KRas* ^*G12D/+*^ *; Trp53* ^*R172H/+*^ *; Atg7* ^*−/−*^ (=KPC7^−/−^)) in which PDAC would then evolve from autophagy-deficient pancreatic tissue (Figure 1A) (Komatsu et al., 2005). All subsequently described data was independent of gender. Surprisingly, Kaplan-Meier survival analysis revealed that autophagy blockage did not change overall survival compared to the autophagy-proficient situation. The mortality rate of 100% was identical and the mean survival was comparable (Figure 1B). However, we noticed that, unlike KPC animals, only a subset of KPC7^−/−^ mice developed PDAC (Figure 1C). Multiple serial sections were performed to rule out microscopic tumors. Just like the overall survival, the survival time stratified for PDAC did not differ between both groups (Figure 1D). Tumor histology was comparable regardless of *Atg7* status (Figure 1E). The presence or absence of autophagy was confirmed by immunohistochemistry for ATG7, LC3, SQSTM1/P62 in strict adherence to published guidelines and procedures (Figure 1E) (Ladoire et al., 2012; Rosenfeldt et al., 2012; Klionsky et al., 2021). As expected, in autophagy-deficient tumors, ATG7 was absent, the LC3 staining pattern was largely homogenous as opposed to the punctate pattern in autophagy proficient tumors and SQSTM1/P62 accumulated (Figure 1E). Human PDAC frequently disseminates and the KPC model has been reported to be metastatic (Morton et al., 2010). Depending on model system and context, autophagy can promote or suppress metastases (Dower et al., 2018). We found micrometastases, in regional lymph nodes, the liver, and the lungs in a small subset of KPC and KPC7^−/−^ mice (Figure 1F). While the metastasis rate in our study was generally low, there was possibly a trend towards a reduced metastatic rate in the tumor-bearing KPC7^−/−^ cohort.

**FIGURE 2:**
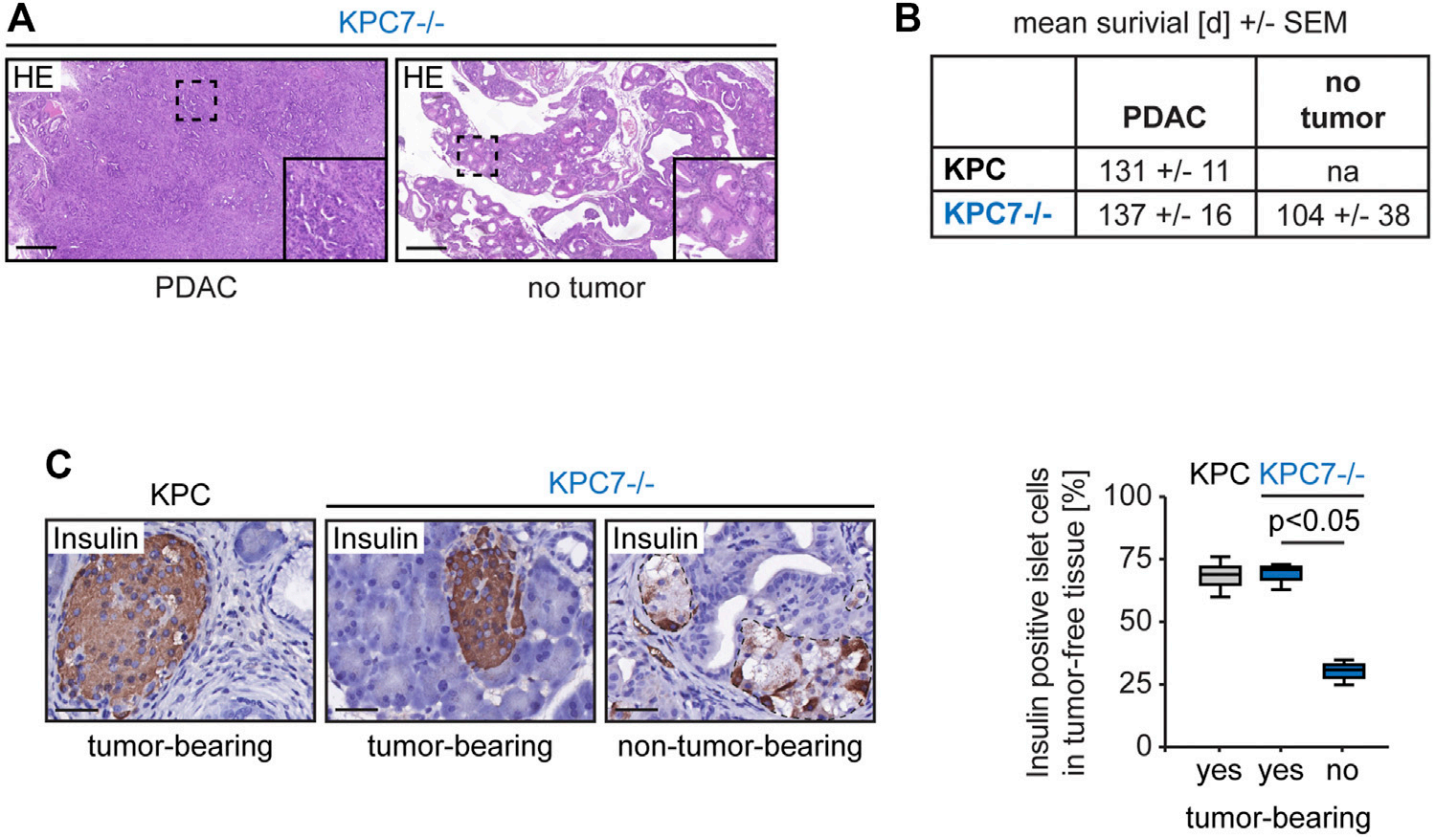
Altered pancreas morphology and reduced percentage of insulin expressing cells in non-tumor-bearing KPC7^−/−^ mice. **(A)** Representative histology as indicated. Scale bars represent 1,000 µm. **(B)** Survival times as indicated. **(C)** Representative immunohistochemistry for Insulin in islets of Langerhans and quantification as indicated. Scale bars represent 40 µm. Statistics: Welch’s *t*-test (n from left to right: 6/3/3).

As mentioned before KPC7^−/−^ mice had a mortality rate of 100% but developed PDAC in only a subset of cases. Histological analysis revealed that non-tumor-bearing KPC7^−/−^ animals featured a completely remodeled pancreas with innumerous low-grade PanINs (Figure 2A). The mean survival time of tumor-bearing and non-tumor-bearing KPC7^−/−^ mice was similar (Figure 2B). Our observations were similar to changes that have previously been observed in *Pdx1-Cre; KRas* ^*G12D/+*^ *; Atg7* ^*−/−*^ mice, with at least one wild-type *Trp53* allele (Rosenfeldt et al., 2013; Yang et al., 2014). Likewise, these animals accumulate PanINs and in addition have a delayed (one wild-type *Trp53* allele) or completely abolished (two wild-type *Trp53* alleles) PDAC onset. We therefore wanted to draw conclusions about the status of the remaining wild-type *Trp53* allele in KPC7^−/−^ animals.

Targeted deep sequencing covering the exonic regions of *Trp53* was carried out to address the issue of wild-type *Trp53* in tumor- and non-tumor-bearing KPC7^−/−^ mice (three animals of each group). Since it is known that KPC animals eventually lose wild-type *Trp53* during PDAC formation, two KPC mice were included as control (Hingorani et al., 2005). DNA was isolated from neoplastic tissue of all cohorts (KPC and tumor-bearing KPC7^−/−^: PDAC tissue, non-tumor-bearing KPC7^−/−^: pancreatic tissue with an abundance of PanINs). Allele frequencies for the c.515G > A mutation (p.R172H) ranged between 47 and 83% and the cellularity of the neoplastic cells was between 10–70%. In every individual sample the frequency of the *Trp53* mutation was higher than the cellularity of the neoplastic cells (data not shown). This suggests that in KPC7^−/−^ mice the wild-type *Trp53* allele is lost not only in PDACs from KPC and KPC7^−/−^ animals, but also in PanINs of the non-tumor-bearing KPC7^−/−^ group.

Insulin is the main hormonal regulator of serum glucose levels and is produced in the islets of Langerhans. Inhibition of *Atg7* in islet cells has been demonstrated to cause fatal hyperglycaemia (Rosenfeldt et al., 2013; Karsli-Uzunbas et al., 2014). Notably, the pancreas morphology of non-tumor-bearing KPC7^−/−^ mice mirrors that of *Pdx1-Cre; KRas* ^*G12D/+*^ *; Trp53* ^*+/+*^ *; Atg7* ^*−/−*^ that are known to develop hyperglycemia (Rosenfeldt et al., 2013). Therefore, we hypothesized that non-tumor-bearing animals suffered from a dysregulated glucose metabolism. Indeed, insulin producing cells were drastically reduced in islets in tumor-free KPC7^−/−^ animals compared to islets in tumor-free tissue regions adjacent to PDACs in KPC and KPC7^−/−^ mice (Figure 2C).

## Discussion

In mice, tumor suppressors such as *Trp53* modulate the effect of autophagy in PDAC development (Rosenfeldt et al., 2013; Mainz and Rosenfeldt, 2018). In the presence of *Trp53*, autophagy blockage via deletion of either *Atg5* or *Atg7* suppresses PDAC formation, but paradoxically increases the formation of low-grade PanINs with impaired capacity to progress to high-grade lesions (Rosenfeldt et al., 2013; Yang et al., 2014). In stark contrast, in the complete absence of *Trp53*, autophagy blockage accelerates tumor formation (Rosenfeldt et al., 2013). Typically, *TP53* is inactivated through missense mutations of one allele and subsequent loss of the other allele in the vast majority of human PDACs as opposed to eventually bi-allelic loss in mice (Saiki and Horii, 2014). TP53 mutants possess disease-promoting properties that differ from the consequences of complete TP53 loss (Sabapathy and Lane, 2018). We now have investigated PDAC development in the context of mutant *Trp53* to address this clinically highly relevant issue. Like the aforementioned studies, we used a mouse model in which genetic recombination occurs in healthy embryonic pancreatic tissue (Gannon et al., 2000; Morton et al., 2010; Rosenfeldt et al., 2013; Yang et al., 2014). We report the following observations. Overall survival time was similar in KPC and KPC7^−/−^ cohorts and the death rate was 100%. In the context of one mutant *Trp53* allele PDAC developed in the absence of *Atg7*, but at a lower frequency compared to *Atg7*-proficency. Non-tumor-bearing KPC7^−/−^ animals featured an overabundance of pre-malignant PanIN lesions and reduced insulin producing cells in the islets of Langerhans. Tumor-bearing animals formed metastasis regardless of *Atg7* status.

By introducing mutant *Trp53* (*Trp53* ^*R172*^) into the equation our data acknowledge an important aspect of human PDAC biology and add further complexity to the interplay between *Atg7* inhibition and *Trp53* status in tumorigenesis. A targeted sequencing approach provided evidence that the wild-type *Trp53* allele is lost in neoplastic tissue of non-tumor-bearing and tumor-bearing KPC7^−/−^ mice. In other words, there was no evidence that a putative retention of the wild-type *Trp53* allele in PanINs was responsible for the emergence of a tumor-free KPC7^−/−^ subgroup.

Alternatively, a special characteristic of *Pdx1-Cre*-mediated recombination potentially could be the cause for the occurence of two subgroups in the KPC7^−/−^ cohort. *Pdx1-Cre*-mediated recombination is known to be generally mosaic regardless of the recombined genetic regions and varies within individual animals (Gannon et al., 2000). Furthermore, it is noteworthy that KPC animals not only experience mosaic recombination but also lose the majority of KRAS (G12D) expressing, and thus recombined cells in the first weeks after birth (Morton et al., 2010). Non-tumor-bearing KPC7^−/−^ pancreata were completely remodelled and packed with low-grade PanINs and regular appearing pancreatic parenchyma was essentially absent. On the other hand, the vast majority of tumor-bearing animals (KPC and KPC7^−/−^) retained some structurally normal pancreas. It is conceivable that non-recombined tissue provides a framework that permits tumor formation from recombined tissue. In such a scenario a drastically effaced pancreatic architecture then no-longer could provide the structural grounds required for tumor development. Supporting the theory that the amount of the retained normal pancreatic tissue is responsible for the dichotomy of the KPC7^−/−^ cohort is the fact that the islets of Langerhans were unremarkable with ample insulin expressing cells in tumor-free tissue adjacent to tumors in KPC and KPC7^−/−^ mice. In contrast, the islets of Langerhans situated within the structurally altered non-tumor bearing KPC7^−/−^ pancreas had a drastically reduced percentage of insulin expressing cells. Insulin is the main hormonal regulator that lowers blood glucose levels. Deletion of *Atg7* has been shown to cause profound destruction of endocrine pancreatic tissue and fatal hyperglycaemia (Quan et al., 2013; Rosenfeldt et al., 2013; Karsli-Uzunbas et al., 2014). Therefore, it is feasible to conclude that the cause of death in non-tumor-bearing KPC7^−/−^ mice is a deregulated glucose metabolism. It must be mentioned that while it is plausible that the ratio of recombined to non-recombined tissue could be the underlying cause for the differences within the KPC7^−/−^ cohort, it is experimentally not proven. It will be important to test this hypothesis and to learn if mosaicism alone and/or an as yet unidentified interplay between *Trp53* and *Atg7* that possibly prevents the clearance of recombined cells are responsible, but this is beyond the scope of this report.

KPC mice have previously been reported to develop metastases with a frequency of approx. 60–70% (Morton et al., 2010). We did not detect grossly evident metastases but found micrometastases at a lower frequency and a possible trend towards a reduced metastatic rate in KPC7^−/−^ animals. It is clearly premature to conclude that autophagy deletion in the context of mutant *Trp53* decreases metastases. The discrepancy between the reported metastatic rate and the low occurrence of disseminated disease in our study is possibly a consequence of our experimental animals being of C57BL/6 background, whereas others have used mice with a mixed background (Morton et al., 2010).

While in our study genetic recombination and therefore the tumor initiating events occurred in healthy embryonic tissue, it is clearly essential to learn what happens if tumor formation commences in adult tissue. Likewise, our study is not an intervention study that investigates the impact of autophagy ablation in an established, autophagy-competent tumor, but rather a prevention study. Recently, mouse models have been developed that potentially allow autophagy inhibition in established tumors via expression of an inducible dominant-negative form of ATG4B (ATG4B(C74A)) or a shRNA against *Atg5* (Cassidy et al., 2018; Yang et al., 2018). In this regard, ATG4B(C74A) has been demonstrated to induce tumor regression in PDAC bearing mice (Yang et al., 2018). Furthermore, it remains to be determined to what extent pre-clinical mouse studies can be transferred to the treatment of patients as compounds that specifically target ATGs are not yet approved for the clinical setting (Piffoux et al., 2021). It is also important to learn how autophagy blockage in the context of mutant *Trp53* shapes metabolism, the tumor stroma and the immune response, all of which have been shown to be directly affected by autophagy (Sousa et al., 2016; Kimmelman and White, 2017; Yamamoto et al., 2020).

In summary, our study adds to the already complex interplay between autophagy inhibition and *Trp53* status in PDAC development and highlights that the genetic background profoundly influences how autophagy blockage affects *de novo* tumorigenesis.

## Data Availability

The original contributions presented in the study are included in the article/Supplementary Material, further inquiries can be directed to the corresponding author.
